# Psychometric properties of the GAD-7 (General Anxiety Disorder-7): a cross-sectional study of the Peruvian general population

**DOI:** 10.1186/s40359-024-01688-8

**Published:** 2024-04-02

**Authors:** David Villarreal-Zegarra, Rubi Paredes-Angeles, Nikol Mayo-Puchoc, Enoc Arenas-Minaya, Jeff Huarcaya-Victoria, Anthony Copez-Lonzoy

**Affiliations:** 1 Universidad César Vallejo, Escuela de Medicina, Trujillo, Peru; 2 Escuela Profesional de Medicina Humana, Universidad Privada San Juan Bautista, Filial Ica, Ica, Peru; 3 Universidad San Ignacio de Loyola, Lima, Peru; 4 Instituto Peruano de Orientación Psicológica, Lima, Peru; 5 Universidad Nacional Mayor de San Marcos, Lima, Peru

**Keywords:** Anxiety, Peru, Psychometrics, Population

## Abstract

**Background:**

Anxiety disorders are among the main mental health problems worldwide and are considered one of the most disabling conditions. Therefore, it is essential to have measurement tools that can be used to screen for anxiety symptoms in the general population and thus identify potential cases of people with anxiety symptoms and provide them with timely care. Our aim was to evaluate the psychometric properties of the General Anxiety Disorder-7 scale (GAD-7) in the Peruvian population.

**Method:**

Our study was a cross-sectional study. The sample included people aged 12 to 65 years in Peru. Confirmatory factor analysis, analysis of measurement invariance, convergent validity with the Patient Health Questionnaire-9 (PHQ-9) and internal consistency analysis were performed.

**Results:**

In total, 4431 participants were included. The one-factor model showed the best fit (CFI = 0.994; TLI = 0.991; RMSEA = 0.068; WRMR = 1.567). The GAD-7 score showed measurement invariance between men and women and between age groups (adults vs. adolescents) (ΔCFI < 0.01). The internal consistency of the one-factor model was satisfactory (ω = 0.90, α = 0.93). The relationship between depressive symptoms (PHQ-9) and anxiety symptoms (GAD-7) presented a moderate correlation (*r* = 0.77).

**Conclusions:**

Our study concluded that the GAD-7 score shows evidence of validity and reliability for the one-factor model. Furthermore, because the GAD-7 score is invariant, comparisons can be made between groups (i.e., by sex and age group). Finally, we recommend the use of the GAD-7 for the general population in the Peruvian context.

## Introduction

Anxiety disorders (ADs) are among the main mental health problems worldwide and are considered among the most disabling mental health problems; they were among the 25 leading causes of mental health burden worldwide in 2019 [1]. The number of ADs has been increasing; in 2015, the estimated incidence of anxiety disorders worldwide was 3.6% (264 million), with a greater proportion of women [2]. The region of the Americas represented 21% (57.22 million) of all cases, and in Peru, 5.7% of people had AD [2]. In 2020, before COVID-19, the estimated global incidence of AD reached 298 million, and after the pandemic, the incidence increased by 25.6%, reaching an estimated global prevalence of 374 million. This increase was also greater for women (27.9%; 51.8 million) than for men (21.7%; 24.4 million). In addition, these percentages vary according to country, with an increase greater than 36.4% in AD occurring in Peru [3].

In this sense, it is necessary to have instruments with good psychometric properties that are brief and screening for easy, fast, and timely risk assessment of this disorder in the population. The most common instruments for measuring anxious symptoms include the Generalized Anxiety Disorders-7 (GAD-7); Beck Anxiety Inventory (21 items) [4]; the Hospital Anxiety and Depression Scale-Anxiety Subscale (7 items) [5]; the Depression, Anxiety and Stress Scale-Anxiety Subscale (7 items) [6]; State-Trait Anxiety Inventory (20 items) [7]; and the Zung Self-rating Anxiety Scale (20 items) [8]. These instruments are most commonly used with adults and adolescents, as older adults have different criteria for anxiety [9].

The GAD-7 is one of the instruments with the fewest number of items and was created according to diagnostic criteria from the Diagnostic and Statistical Manual for Mental Disorders, Fourth Edition (DSM-IV), to detect generalized anxiety disorders [10]. Additionally, it is widely used in the clinical field [11, 12], demonstrating good performance and adequate diagnostic accuracy [11]. Similarly, this instrument has shown good results in different populations and situations, such as in university students [13, 14], adolescents [15], older adults [16], and health workers [17]; in virtual evaluations [18]; and because it is used for screening, it is also useful for obtaining prevalence estimates in the general population [19, 20].

Despite being widely used, the GAD-7 has some heterogeneity in terms of its dimensionality. Most studies agree that the original one-factor model works well [16, 21–23]; however, some studies report some modifications to this factorial structure, considering errors correlated between somatic items [24, 25]. Other studies have considered a two-factor model, distinguishing cognitive-emotional aspects from somatic ones [26, 27], and another study has suggested using a second-order model, taking cognitive-emotional and somatic elements as first-order factors [28]. However, to date, there is no consensus on the most appropriate factor structure for the GAD-7, but the one-factor model is the most widely used [35, 36].

Another important property is measurement invariance, understood as the equivalence (in psychometric terms) of a construct across groups, which has the same meaning as those groups and is a prerequisite for comparing group means [29]. This property is not always reported, and the results of measurement invariance studies of GAD-7 scores according to age and sex have some discrepancies. Some studies have shown that invariance is achieved by sex and age [26], while in other studies, invariance is violated [30]; therefore, if this property is not verified in a population, comparisons between sex or age groups can lead to biased results and interpretations. Despite the available evidence, there are gaps in the knowledge of which variables GAD-7 is invariant and which are not. Therefore, this is still an open area of research.

Additional evidence of validity reported for the GAD-7 is its relationship with other variables, which are strongly related to depressive symptoms and are generally measured by the PHQ-9 [24, 28, 31]. This relationship is consistent with what is expected between depressive and anxiety disorders, both of which are considered common mental disorders due to their high prevalence and comorbidity [2].

The GAD-7 is a widely researched and useful tool for detecting potential cases of anxiety symptoms. Despite its usefulness, evidence regarding its factor structure and its invariance between groups is mixed. This highlights the need for further research to clarify these aspects. Given the importance of confirming adequate psychometric properties before using an instrument in a specific population, our study aims to: (1) Analyze the factorial structure of the original GAD-7 in the Peruvian population; (2) Evaluate GAD-7 measurement invariance based on sex and age; (3) Report the relationship with other variables (depressive symptoms); and (4) Estimate the reliability of the GAD-7. Our central hypotheses are that the GAD-7 has a strong factor structure, is invariant across gender and age groups, has a strong relationship with depressive symptoms, and has optimal levels of reliability.

## Methods

### Study design

Secondary data from six studies were obtained before and during the COVID-19 pandemic, and a cross-sectional design was used to evaluate the psychometric properties and validity of the GAD-7 in teenagers and adults in Lima, Peru.

### Setting

Peru is a middle-income Latin American country that has had several problems in its health system since before the outbreak of COVID-19. The Peruvian government decreed of a state of sanitary emergency (March 16, 2020) to mitigate the spread of the infection, and a suppressive strategy was adopted (social isolation or quarantine). Moreover, the suspension of activities such as economic, academic, transport, and recreational activities was stipulated, and only essential activities related to the supply of products and services for public health were maintained [32, 33].

Evidence indicates that the mental health impacts (e.g., anxiety, depression, posttraumatic stress) caused by strict health measures in low-income and middle-income countries have a significant mental impact, which contributes to a slow recovery toward normality [34].

### Participants

The following six datasets were used to analyze the data of patients who met the inclusion criteria: (1) aged 12 years to 65 years. (2) Patients had complete data on the GAD-7 score, sex, and age. (3) Participants must have agreed to participate in the study first after providing informed consent. For those under 18 years of age, only those participants whose parents provided consent for their children to participate were considered (informed consent). We excluded participants with implausible data (i.e., age > 99 years). Nonprobabilistic sampling was performed for all the datasets.

### Measurement

#### Anxiety symptoms

The General Anxiety Disorder-7 scale (GAD-7) is a 7-item self-report Likert scale that was developed to assess the severity of anxiety disorders based on the Diagnostic and Statistical Manual of Mental Disorders, 4th edition (DSM-IV). This self-report measures the indicators of anxiety symptomatology in the last 2 weeks. Each item is rated on a 4-point Likert-type scale (0 = not at all; 1 = several days; 2 = more than half the day; 3 = nearly every day) [10]. To identify possible cases of general anxiety disorder (GAD), some studies considered using a cutoff range of 10 points because this cutoff provides a high balance between sensitivity and specificity [35, 36]. We use the Spanish version of GAD-7 by Soto-Balbuena and collaborators [22].

#### Depressive symptoms

The Patient Health Questionnaire-9 (PHQ-9) is a 9-item Likert scale developed to measure the severity of depressive symptoms; this scale was designed from the nine diagnostic criteria from the DSM-IV. The instrument reports the indicators of depressive symptomatology over the past 2 weeks. Its response options were 4-point Likert-type scales (0 = not at all; 1 = several days; 2 = more than half the days; 3 = nearly every day) [37]. According to other studies, a standard cutoff score of 10 or above can be used for screening to detect moderate depressive symptoms [37, 38]. The PHQ-9 has been validated in a Peruvian population sample, where it presented optimal validity and reliability values [39].

### Procedure

Participants were recruited through an online Google Forms form, which was distributed to potential participants through networking via instant messaging applications such as WhatsApp and Telegram, as well as social media platforms such as Facebook and Instagram. Participants received no economic incentives or rewards. Participation was voluntary, and they accepted informed consent before the evaluation process began.

### Statistical methods

All the analyses were performed in RStudio [40] using the packages lavaan [41], semTool [42] and semPlot [43].

#### Descriptive analysis

A descriptive analysis of participant characteristics was also conducted (mean, standard deviation, percentage, and frequency). The prevalence of anxious and depressive symptoms was based on the cut-off of 10 points or more for the GAD-7 and PHQ-9, respectively. In addition, we performed a descriptive analysis of the items using mean, standard deviation, skewness, and kurtosis.

#### Confirming factor analysis

We used one-factor, two-factor, and second-order factor models to assess the factorial structure of GAD-7 scores. All the models use the weighted least squares means and variance adjusted (WLSMV) estimator because of its ability to provide a good option for modeling categorical or ordered data [44, 45]. Additionally, a polychoric correlation matrix was calculated. Therefore, to evaluate the model fit, the weighted root mean square residual (WRMR), the comparative fit index (CFI), and the root mean square error of approximation (RMSEA) along with 90% confidence intervals (90% CIs) were used. A reasonably good fit is recommended following the following criteria: (a) WRMR < 1 or below; (b) RMSEA < 0.08 or below; and (c) CFI and TLI > 0.95 or above [46, 47]. This analysis was performed to determine the best factor structure of the GAD-7.

#### Invariance between groups

Testing for measurement invariance involves testing a series of hierarchically nested models to assess whether the instrument is stable between two or more groups; thus, comparisons can be made between them [48]. Comparisons were made between sex groups (male and female) and ages (adolescents and adults). To compare models with more restrictions against models with fewer restrictions, we used ΔCFI and ΔRMSEA as variants of the comparative fit index and the root means the square error of approximation, respectively. Thus, ΔCFI values < 0.01 and ΔRMSEA values < 0.015 provide evidence for measurement invariance [29, 49]. In addition, we assessed other fit indices, such as the CFI and RMSEA, along with 90% confidence intervals. This analysis was performed to determine whether the GAD-7 showed measurement invariance between groups, allowing comparisons to be made between these groups.

#### Convergent validity

To examine convergent validity, the GAD-7 and the PHQ-9 total scores were correlated. Due to its concordance with other samples, the GAD-7 score was hypothesized to be strongly correlated with depression indicators (PHQ-9) [24, 28, 31]. This correlation was determined by Pearson’s r (r). A large (*r* > 0.70), moderate (*r* > 0.50) or small (*r* > 0.30) ratio was determined based on the size of the correlation coefficient [50].

#### Reliability

Internal consistency analyses were performed using two coefficients: the ordinal alpha (α) and categorical omega (ω) coefficients. Both are acceptably reliable when the coefficient values are greater than 0.80 [50]. In addition, we performed a test item correlation analysis.

### Ethics aspects

The institutional research ethics committee of the Instituto Peruano de Orientación Psicológica approved the study protocol.

## Results

### Characteristics of the participants

Initially, we found 5048 records in the different datasets, and we eliminated 617 records after applying the inclusion criteria (12.2%). Therefore, the study included a total sample of 4431 participants. The sample consisted of 1929 men (43.5%) and 2502 women (56.5%), and the ages ranged from 11 to 65 years (M = 28.9 years; SD = 12.8). Furthermore, 3581 were adults (80.8%), and 850 were adolescents (19.2%). Additionally, 3653 patients were evaluated during the COVID-19 pandemic (82.4%), and 778 were evaluated before the pandemic (17.6%). In terms of prevalence, we found that 20.8% of participants presented anxious symptoms (*n* = 922) and that 29.5% had depressive symptoms (*n* = 1307). In addition, the raw scores of the GAD-7 and their measures of skewness and kurtosis are presented (see Table 1).

**Table 1:** Descriptive statistics and item‒test correlations for the items on the general anxiety disorder-7 (*n* = 4431)

Items	M	SD	g^1^	g^2^	r_it_
1. Feeling nervous, anxious, or on edge [Se ha sentido nervioso(a), ansioso(a), o con los nervios de punta]	0.83	0.87	0.90	0.21	0.83
2. Not being able to stop or control worrying [No ha sido capaz de parar o controlar su preocupación]	0.64	0.85	1.29	1.06	0.86
3. Worrying too much about different things [Se ha preocupado demasiado por motivos diferentes.]	0.97	0.89	0.73	-0.07	0.86
4. Trouble relaxing [Ha tenido dificultad para relajarse]	0.90	0.90	0.80	-0.08	0.85
5. Being so restless that it is hard to sit still [Se ha sentido tan inquieto(a) que no ha podido quedarse quieto(a)]	0.58	0.81	1.39	1.37	0.80
6. Becoming easily annoyed or irritable [Se ha molestado o irritado fácilmente]	0.98	0.91	0.69	-0.24	0.74
7. Feeling afraid as if something awful might happen [Ha tenido miedo de que algo terrible fuera a pasar]	0.86	0.92	0.91	0.05	0.76

*Notes* M = mean; SD = standard deviation; g^1^ = skewness; g^2^ = kurtosis; r_it_ = item-test correlation. The Spanish version of the items is presented in square brackets

### Confirmatory factor analysis

Our study evaluated different factor models based on previous studies. Based on this, we determined that all the models evaluated achieved optimal goodness-of-fit indices (see Table 2). The model with two correlated factors exhibited a very high correlation (Φ > 0.90). Therefore, we believe that both factors overlap, which means that it is not considered a parsimonious model and should be discarded. According to the second-order models, the two specified factors had loads very close to one concerning their general factor. This is why it is not considered a stable model, since both specific dimensions can actually be part of a one factor model.

**Table 2:** Goodness-of-fit indices of the tested models (*n* = 4431)

	χ^2^ (df)	CFI	TLI	RMSEA (CI 90%)	WRMR
One-factor	304.6 (14)	0.994	0.991	0.068 (0.062–0.075)	1.567
Two-factors	187.7 (13)	0.997	0.994	0.055 (0.048–0.062)	1.210
Second order with two-factors	71.8 (12)	0.999	0.998	0.034 (0.026–0.041)	1.210

*Notes Χ*^2^ = chi-square; df = degrees of freedom; CFI = comparative fit index; RMSEA = root mean square error of approximation. WRMR = weighted root mean square residual. TLI = Tucker–Lewis index

**Fig. 1:**
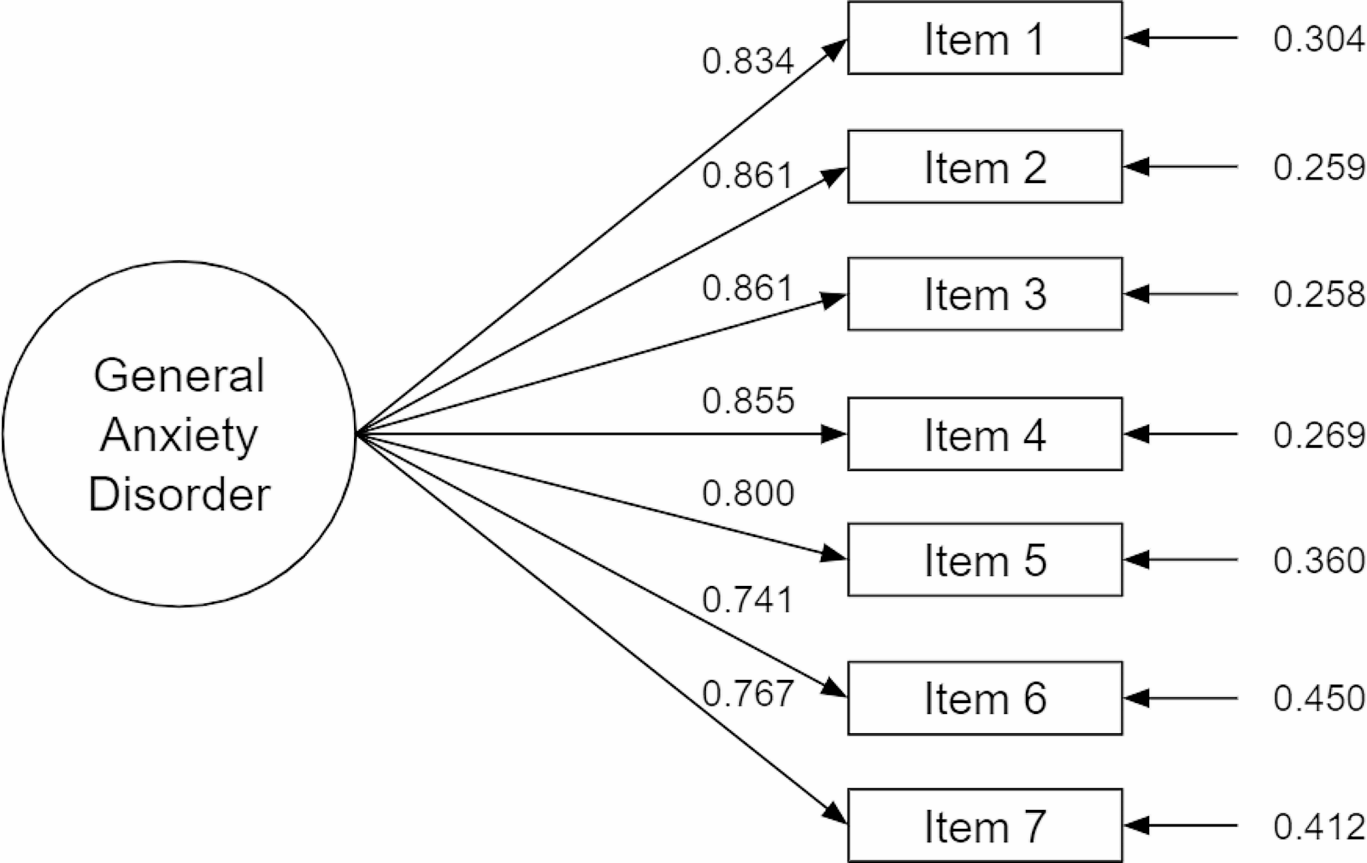
One-factor model of the GAD-7 score

Our study considers the one-factor model more appropriate because it is more parsimonious and requires fewer assumptions. In addition, all factor loadings were greater than 0.71 (see Fig. 1). This decision was made because the other two models present overlap and the one-factor model is the most used and stable model found in other studies.

### Invariance between groups

Measurement invariance analysis between sex and age revealed that both groups were invariant, so comparisons could be made between each of these groups. Total scores can be compared between males and females or between age groups (adults vs. adolescents) as the ΔCFI and ΔRMSEA values remain at appropriate levels (see Table 3). In addition, the CFI and RMSEA values remained adequate for the configural, metric, and strong models. The configural level suggested that the different groups (men vs. women, and adults vs. adolescents) presented an equivalent factor structure, i.e., a seven-item, one-dimensional model. The metric level indicated that the different groups had equivalent factor loadings, and the strong level suggested that there were equivalent thresholds between the groups.

**Table 3:** Summary of goodness-of-fit statistics for tested models in multigroup analyses (*n* = 4431)

		Χ^2^	df	CFI	RMSEA	ΔCFI	ΔRMSEA
Sex (men vs. women)	Configural	157.8	28	0.981	0.046		
	Metric	95.6	34	0.990	0.029	0.009	-0.017
	Strong	120.8	40	0.987	0.030	-0.003	0.001
Age (adults vs. adolescents)	Configural	125.5	28	0.985	0.040		
	Metric	113.8	34	0.988	0.033	0.003	-0.007
	Strong	134.6	40	0.986	0.033	-0.002	0.000

*Notes x*^2^ = chi-square; df = degrees of freedom; CFI = comparative fit index; RMSEA = root mean square error of approximation; ΔCFI = variation in the comparative fit index; ΔRMSEA = variation in the root mean square error of approximation

### Reliability

The internal consistency of the one-factor model was satisfactory (ω = 0.90; α = 0.93). The item-test correlation analysis indicated that, even if one item within the GAD-7 were eliminated, the internal consistency coefficient alpha would remain adequate in all cases (see Table 1). Therefore, eliminating one item would not affect the reliability of the scale.

### Convergent validity

A moderate correlation was shown between depressive symptoms and anxiety symptoms (*r* = 0.77), as other studies have shown. Therefore, it can be inferred that the instrument has convergent validity.

## Discussion

### Main findings and interpretation

Our study validates a brief tool for measuring anxious symptoms, which represents a valuable resource for the development of mental health research and a potential screening tool in the primary care setting in Peru. Our study concluded that the GAD-7 score is valid and reliable according to the one-factor model. Internal structure validity evidence for the GAD-7 suggests that its seven items can be summed to obtain a total score. In addition, our study revealed that comparisons can be made between groups according to factors such as sex and age. For example, a comparison of GAD-7 scores can be made between men and women. Similarly, evidence of convergent validity indicates that the GAD-7 score in the Peruvian context is strongly related to depressive symptoms, which has been found in different studies, suggesting that the instrument behaves consistently with other studies. Finally, the GAD-7 score for the one-factor model presented optimal reliability values.

### Confirmatory factor analysis

Our study revealed that the GAD-7 score has one factor. This finding is consistent with results reported in previous studies [21, 23, 51]. Other studies have suggested two-factor or higher-order models, but these results are not necessarily contradictory because these highly related dimensions are part of the same overall construct, anxiety symptoms [27]. Therefore, although somatic and cognitive-emotional factors are theoretically valid, they do not seem to be distinguishable at the empirical level in the general population [27].

A one-factor model indicates that the GAD-7 can use a total score and establish cutoff points with sensitivity and specificity values [36]. In addition, a Peruvian study also found adequate levels of sensitivity and specificity for GAD-7 with a cut-off of 10 points or more [52]. However, for models with two or more factors, sensitivity and specificity analyses must be performed for each factor. We did not find any sensitivity or specificity studies for the two-factor models of the GAD-7 score. Therefore, the one-factor model is more commonly used and studied.

### Invariance between groups

Our results showed invariance of the GAD-7 score across sex and age. Although few studies have explored the invariance of the GAD-7 score, similar findings were obtained by [26], who found invariance across sex, age and marital status, level of education, and employment situation in Spanish primary care patients. Likewise, another study reported invariance regarding sex, strata, and linguistic background in a sample of patients after traumatic brain injury [23]. A study that included a Peruvian sample also reported invariance of the GAD-7 score and other short versions across sexes; however, only university students were considered [51]. The interpretation of our results suggests that, for the different groups, participants perceive the existence of a single factor consisting of seven items (configural invariance), indicating that the items have equivalent factor loadings, and therefore the items contribute equally to the construct (metric invariance). In addition, the thresholds of these items show equivalent values across groups, allowing for comparisons between groups (strong invariance) [29].

### Convergent validity

At the level of convergent validity, the GAD-7 score showed a moderate correlation with the PHQ-9 score, which measures depressive symptoms. These findings are consistent with the results of several studies that have shown a direct relationship between moderate and strong strength [23, 24, 27, 31]. At the level of reliability, other studies have also shown that the GAD-7 has adequate internal consistency values for one-factor models [24, 31, 53].

### Public health implications

In Peru, there are no clinical practice guidelines for the assessment, diagnosis or treatment of anxiety disorders. Our study allows the GAD-7 to be used as a scale to detect depressive symptoms in the general population. Because of its brevity, we recommended their use in future Peruvian clinical practice guidelines on anxiety from the Ministry of Health or Social Health Insurance (EsSalud). Considering that there are currently a study evaluating the sensitivity and specificity of different cut-offs for GAD-7 in the Peruvian population [52].

### Strengths and limitations

The main strength of our study is the large sample size. Our study has several limitations. First, our study was not probability-based, so it cannot be generalized to other populations. Second, our study does not propose a cutoff point for determining whether participants have anxiety symptoms. Third, it was not possible to assess invariance with other groups of interest, such as marital status, chronic illness or economic status.

### Conclusions and recommendations

Our study concluded that the GAD-7 score shows evidence of validity and reliability for the one-factor model. Furthermore, because the GAD-7 score is invariant, comparisons can be made between groups (i.e., by sex and age group). Finally, we recommend the use of the GAD-7 for the general population in the Peruvian context.

## Data Availability

Access to data will only be by convincing request.To review the manuscript we have enabled a link to our dataset https://zenodo.org/records/10600793.
